# A Polyphasic Approach to Compare the Genomic Profiles of Aflatoxigenic and Non-Aflatoxigenic Isolates of *Aspergillus* Section *Flavi*

**DOI:** 10.3390/toxins12010056

**Published:** 2020-01-16

**Authors:** Asmaa Abbas, Taha Hussien, Tapani Yli-Mattila

**Affiliations:** 1 Department of Biochemistry, University of Turku, FI-20014 Turku, Finland; asmaa.abbas@utu.fi (A.A.); taha_bio@yahoo.com (T.H.); 2 Department of Chemistry, Faculty of Science, Sohag University, Sohag 82524, Egypt; 3 Department of Food Toxicology and Contaminant, National Research Center, Cairo 12311, Egypt

**Keywords:** *Aspergillus*, aflatoxins, HPLC, genetic diversity, RAPD, ISSR, sequencing

## Abstract

Aflatoxins (AF) are highly toxic compounds produced by *Aspergillus* section *Flavi*. They spoil food crops and present a serious global health hazard to humans and livestock. The aim of this study was to examine the phylogenetic relationships among aflatoxigenic and non-aflatoxigenic *Aspergillus* isolates. A polyphasic approach combining phylogenetic, sequence, and toxin analyses was applied to 40 *Aspergillus* section *Flavi* isolates collected from eight countries around the world (USA, Philippines, Egypt, India, Australia, Indonesia, China, and Uganda). This allows one to pinpoint the key genomic features that distinguish AF producing and non-producing isolates. Based on molecular identification, 32 (80%) were identified as *A. flavus,* three (7.5%) as *A. parasiticus,* three (7.5%) as *A. nomius* and one (2.5%) as *A. tamarii.* Toxin analysis showed that 22 (55%) *Aspergillus* isolates were aflatoxigenic. The majority of the toxic isolates (62.5%) originated from Egypt. The highest aflatoxin production potential was observed in an *A. nomius* isolate which is originally isolated from the Philippines. DNA-based molecular markers such as random amplified polymorphic DNA (RAPD) and inter-simple sequence repeats (ISSR) were used to evaluate the genetic diversity and phylogenetic relationships among these 40 *Aspergillus* isolates, which were originally selected from 80 isolates. The percentage of polymorphic bands in three RAPD and three ISSR primers was 81.9% and 79.37%, respectively. Analysis of molecular variance showed significant diversity within the populations, 92% for RAPD and 85% for ISSR primers. The average of Polymorphism Information Content (PIC), Marker Index (MI), Nei’s gene diversity (H) and Shannon’s diversity index (I) in ISSR markers are higher than those in RAPD markers. Based on banding patterns and gene diversities values, we observed that the ISSR-PCR provides clearer data and is more successful in genetic diversity analyses than RAPD-PCR. Dendrograms generated from UPGMA (Unweighted Pair Group Method with Arithmetic Mean) cluster analyses for RAPD and ISSR markers were related to the geographic origin.

## 1. Introduction

*Aspergillus* is a diverse genus that has a high economic and social impact. Species occur worldwide in various environments, which spoil food, produce mycotoxins, and are commonly reported as human and animal pathogens. Aflatoxins (AFs) are one of a group exceedingly toxic secondary metabolites derived from polyketides, generally produced by three main species that belong to the section *Flavi*: *Aspergillus flavus*, *Aspergillus parasiticus*, and *Aspergillus nomius*, which prefer tropical and subtropical environments, with high temperatures, high humidity [1], and oxygen pressure [2]. AFs destroy an estimated 25% or more of the world’s food crops annually [3]. There are approximately 14 types of aflatoxins found in nature but only four of them (AFB1, AFB2, AFG1, and AFG2) are really dangerous for living organisms. AFs occur in various cereals, oilseeds, maize, soil and nuts during pre-harvesting, harvesting or storage conditions [4]. Aflatoxin levels exceeding 0.5–15 ppb in nuts, grains, dried fruits, and milk are strictly prohibited in the World Health Organization [3]. 

In the last decade, the utilization of molecular tools empowered the identification of new species belonging to the *Aspergillus* section *Flavi*, particularly, based on the DNA sequences of the ribosomal gene of the internal transcribed spacer (ITS) region [5]. Moreover, these species can be distinguished through their ability to produce aflatoxins. *A. flavus* produce aflatoxins of B type, however *A. parasiticus* and *A. nomius*, produce both B and G aflatoxin types and *A. tamarii* is non-aflatoxin producer [6,7]. The best way to identify new species and confirm the status of morphological species is to use phylogenetic species recognition, with genealogical concordance, in which several separate DNA sequences are used together (GCPSR) [8]. The sequence data can also be used for designing probes for species-specific genotyping of the isolates and multiplex detection of fungal species [9]. 

PCR based methods that detect the presence or expression of aflatoxin pathway genes have been used as diagnostic tools for aflatoxigenic strains in selected crops [10]. Aflatoxin production by *Aspergillus* spp. requires presence of these genes. Mainly, the presence of regulatory (*aflR* and *aflS*) and some structural (*aflD*, *aflQ*, and *aflP*) genes are tested. A recently published article [11] described the difference between aflatoxigenic and non-aflatoxigenic *A. flavus* based on the molecular analysis of the aflatoxin biosynthesis genes. At least 34 genes have been identified in the aflatoxin biosynthesis pathway gene cluster in *A. flavus* and *A. parasiticus*. A positive regulatory gene *aflR* encodes a sequence-specific, Gal4-type C6-zinc binuclear cluster DNA binding (Zn(II)2Cys6) protein that is required for transcriptional activation of the AF structural genes [12]. 

The molecular markers, random amplified polymorphic DNA (RAPD), restriction fragment length polymorphism (RFLP), amplified fragment length polymorphism (AFLP), and inter simple sequence repeats (ISSR), have been used broadly to assess the genetic variation and polymorphic fragments of a wide range of species [13,14,15,16]. Each marker has advantages and disadvantages. RFLP has a very limited sensitivity of detection and it is difficult to obtain good profiles with trace biological evidence or too aged samples. AFLP can be used for any DNA origin with high detection marker system but it requires high quality of genomic DNA and high-quality electrophoresis system. Being a quick, cheap and sensitive method, RAPD can be applied proficiently to recognize useful polymorphisms. RAPDs are dominant markers, and some loss of information might occur, but storage period of DNA extractions is not critical with RAPDs. The ISSR markers, derived from SSR, amplify a detailed region between two microsatellites. They are informative, efficient and exhibit high polymorphic bands. DNA sequence is amplified using single primer and no prior knowledge of DNA sequence information is required in both RAPD and ISSR markers [17,18,19].

Up to the present time, seven mycotoxigenic *Aspergillus* species, four *Fusarium* species and one *Penicillium* species have been isolated from several agricultural crop commodities in Philippines [20]. However, few studies have been conducted to evaluate the genotypic differences and molecular-based identification methods for *Aspergillus* section *Flavi* isolated from the Philippines [21]. On the contrary, there are many reports dealing with isolation, identification and genetic relationships between *Aspergillus* section *Flavi* isolated from Egyptian crops [22,23,24]. Within this context, the aim of this study was to (i) explore the toxins of *Aspergillus* section *Flavi* isolates using HPLC to differentiate between aflatoxigenic and non-aflatoxigenic isolates, (ii) sequence the ITS region of 26 *Aspergillus* isolates from different geographic origins (for molecular identification) and *aflR* gene (master regulatory transcription factor in the AF pathway) in 14 isolates representing both aflatoxigenic and non-aflatoxigenic isolates and (iii) find genetic relationships and molecular biodiversity among all *Aspergillus* isolates through RAPD and ISSR molecular markers.

## 2. Results

### 2.1. Toxin Analysis

Forty *Aspergillus* isolates from different geographic regions were examined for aflatoxin production using high performance liquid chromatography (HPLC) (Table 1). The aflatoxin quantification in ppb for the *Aspergillus* spp. was calculated according to the retention time and the peak area of an aflatoxin standard solution. Six of the 11 isolates from the SRRC culture collection were AF producers. The highest concentration was produced by AF 2653 (2339.6 ppb B-types AF). Eleven isolates of the 21 isolates from the Philippines were AFs producers, of which 7P and 9P produced all AF types. 9P isolate was found to be the most effective AF producer (14,416.1 ppb). Regarding to the Egyptian isolates, there were five aflatoxin producing isolates among the overall eight isolates and 21E was the most effective AF producer (1157.6 ppb). 

### 2.2. Morphological and Molecular Identification

The isolates were identified morphologically by [21]. For molecular identification, ITS1 and ITS2 regions were sequenced in most isolates and *aflR* gene region was sequenced in the rest of the isolates and compared to known DNA sequences of reliable isolates. The molecular identification, accession numbers and sequencing results for the *Aspergillus* spp. are shown in Table 1.

### 2.3. Genotyping Analysis of ITS Sequences

Thirty *Aspergillus* isolates were identified with 100% sequence similarity to the sequences found in the NCBI GenBank. Nine isolates (AF 2118, AP143, 2P, 25P, 81P, 108P, 110P, 112P and 1E) possessed heterozygous ITS1 and/or ITS2 loci; and displayed ~99% sequence similarity with the GenBank sequences. We deposited the sequences from these nine isolates to the GenBank to record them with new accession numbers. One isolate, 111P had short good sequence which fitted as *A. flavus*. From the 40 isolates used for the genetic relationship, 32 isolates (80%) belonged to *A. flavus*, three isolates (7.5%) belonged to *A. parasiticus*, three isolates (7.5%) belonged to *A. nomius* and one isolate (2.5%) belonged to *A. tamarii*. The Philippines isolates contained 17 *A. flavus*, three *A. nomius* and one *A. tamarii*. All isolates from Egypt belonged to *A. flavus*. The phylogenetic tree for ITS sequences is shown in Figure S1. *A. flavus* isolates formed a big cluster, which was separated from clusters formed by *A. parasiticus, A. nomius* and *A. tamarii*.

### 2.4. Correlation between aflR Gene Profile and Aflatoxin Production

**Figure 1:**
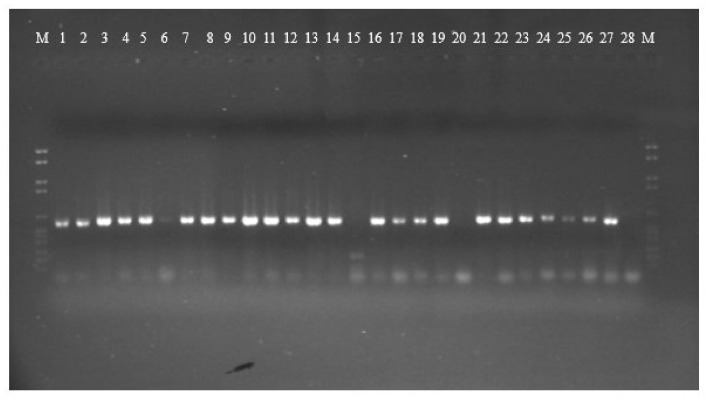
Gel electrophoresis of *afl*R gene amplification for 15 aflatoxigenic and 13 non-aflatoxigenic isolates. Aflatoxigenic isolates are in the following lanes: 1 = 7P, 2 = 9P, 3 = 34P, 4 = 41P, 5 = 42P, 6 = 45P, 7 = 51P, 8 = AF 144, 9 = AF 1554, 10 = AP 2040, 11 = AF 2653, 12 = AP 1311, 13 = 44E, 14 = 3E and 21 = AF 1305. Non-aflatoxigenic isolates are in the following lanes: 15 = 2P, 16 = 3P, 17 = 23P, 18 = 25P, 19 = 58P, 20 =85P, 22 = AF 2041, 23 = AF 2118, 24 = AF 2525, 25 = AF 2649, 26 = 42E, 27 = 45E, and 28 = 30E.

**Figure 2:**
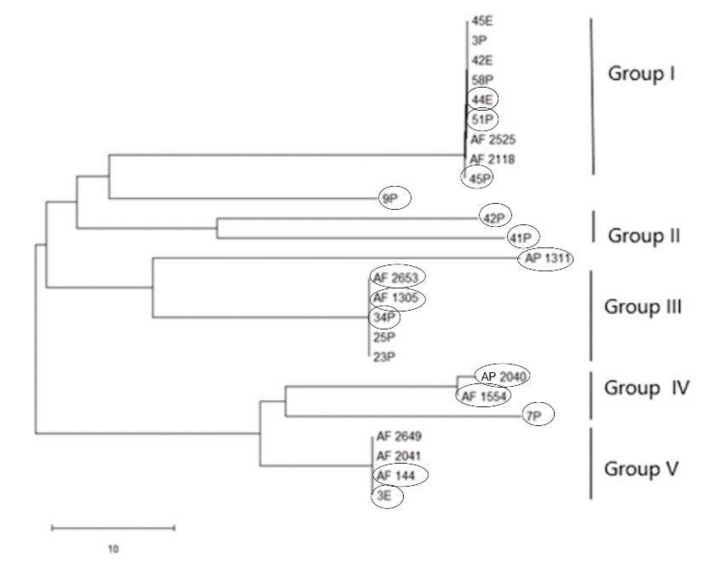
Neighbor-joining tree for 15 aflatoxigenic and 10 non-aflatoxigenic *Aspergillus* isolates based on concatenated partial gene sequences of *afl*R. E isolates are from Egypt and P isolates are from the Philippines. Aflatoxigenic isolates are surrounded by circles.

PCR amplification for *aflR* gene (the master regulator in the AF pathway) was done on 15 aflatoxigenic and 13 non-aflatoxigenic isolates. All of them exhibit amplified products except three isolates; 2P, 85P and 30E. Nevertheless, the aflatoxigenic isolates exhibited more intense bands on the gel than the non-aflatoxigenic ones except 45P which showed faint band. As is clear from Figure 1, lane 17, lane 18, lane 24, lane 25 and lane 26 (23P, 25P, AF 2525, AF 2649 and 42E) show faint bands and are non-aflatoxin producers. Phylogenetic analyses from the concatenated *aflR* sequences divided the twenty-five *Aspergillus* isolates into five distinct supported groups (Figure 2). The first group, consisted of non-aflatoxigenic *A. flavus* isolates except 44E, 51P and 45P which produce B-types AF. The second group, consisted of two *A. flavus* B-producers. The third group consisted of toxigenic *A. flavus* isolates except two non-producers. The fourth group includes aflatoxigenic isolates of which one is *A. flavus,* the second one *A. parasiticus* isolate AP 2040 and the third one *A. nomius* isolate 7P. The fifth group consists of B-types producing isolates except AF 2041 which is non-aflatoxigenic. The second *A. nomius* isolate 9P and the second *A. parasiticus* isolate AP 1311 form their own groups.

### 2.5. RAPD and ISSR Banding Pattern, PIC and MI

**Figure 3:**
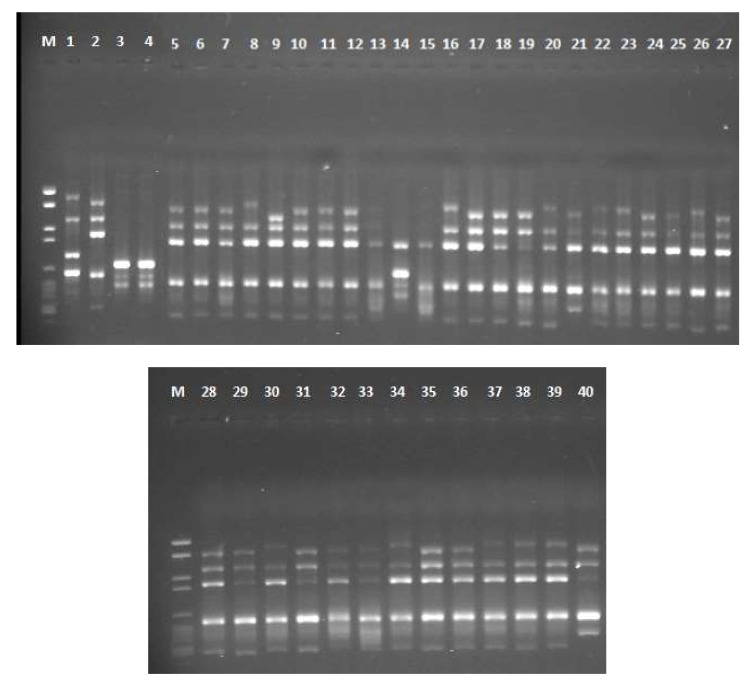
Gel electrophoresis of PCR-ISSR amplification using (GTG) 5 primer for 40 *Aspergillus* isolates. The upper gel contains the following lanes: M = DNA marker, 1 = AF 144, 2 = AF 1305, 3 = AF 1554, 4 = AF 2041, 5 = AF 2118, 6 = AF 2525, 7 = AF 2649, 8 = AF 2653, 9 = AP 143, 10 = AP 1311, 11 = AP 2040, 12 = 2P, 13 = 3P, 14 = 7P, 15 = 9P, 16 = 23P, 17 = 25P, 18 = 32P, 19 = 34P, 20 = 41P, 21 = 42P, 22 = 45P, 23 = 47P, 24 = 51P, 25 = 58P, 26 = 64P and 27 = 81P. The lower gel contains the following lanes: 28 = 85P, 29 = 108P, 30 = 110P, 31 = 111P, 32 = 112P, 33 = 1E, 34 = 3E, 35 = 16E, 36 = 21E, 37 = 30E, 38 = 42E, 39 = 44E and 40 = 45E.

Three RAPD primers were screened for *Aspergillus* spp. A total of 317 clear bands were produced with an average of 106 bands per each primer. The three ISSR primers yielded 506 distinct bands with an average of 169 bands per primer. The number of polymorphic bands produced by each primer ranged from three bands (RAPD primer 5) to eight bands (AGAG)4G primer. Two primers (RAPD 2 and (AGAG) 4G) exhibited 100% polymorphism. The total percentage of polymorphic bands in RAPD and ISSR primers is 81.9% and 79.37% respectively. For RAPD primers, the PIC value ranged from 0.45 (RAPD 5) to 0.76 (RAPD 1) with an average of 0.65. For ISSR primers, the PIC value ranged from 0.77 ((GTG) 5) to 0.81 ((AGAG) 4G) with an average of 0.79. The average of PIC and MI values for ISSR markers (0.79, 0.56) were found to be higher than for RAPD markers (0.65, 0.47) (Table 2). Gel electrophoresis photo for 40 *Aspergillus* isolates amplified with (GTG) 5 primer is shown in Figure 3.

### 2.6. Allelic and Genetic Diversity among Aspergillus Isolates

The parameters, Number of alleles (Na) and Effective number of alleles (Ne) illustrated by RAPD primers (2.00, 1.13) are approximately similar to those of the ISSR primers (2.00, 1.33). On the other hand, Nei’s gene diversity (H) and Shannon’s diversity index (I) of ISSR primers (0.24, 0.408) are higher than those of RAPD primers (0.11, 0.223) (Table 3). 

### 2.7. AMOVA Analysis

Table 4 revealed the AMOVA analysis for *Aspergillus* isolates. The percentage of total variance exemplified significant diversity within the populations, 92 % for RAPD and 85% for ISSR primers, where the *P*-value was ˂ 0.05. But, there was no significant variety among the populations, 8% for RAPD and 15% for ISSR markers, because the *P*-value was ˃ 0.05.

### 2.8. RAPD and ISSR Dendrogram Analyses

**Figure 4:**
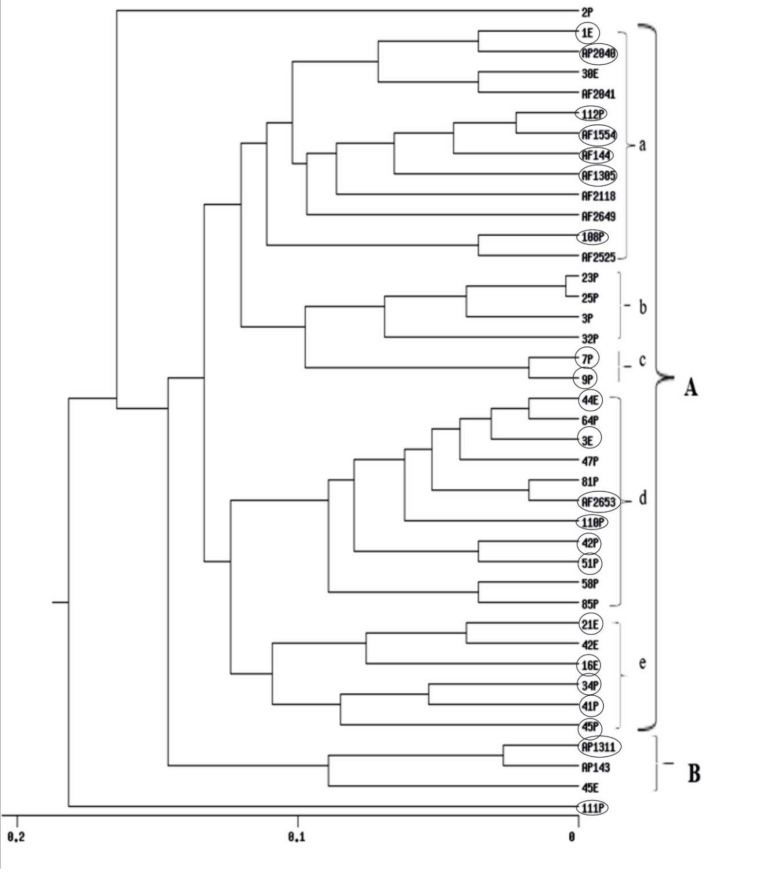
UPGMA cluster analysis generated from RAPD-PCR of 40 *Aspergillus* isolates. E isolates are from Egypt, P isolates are from Philippines, *A. flavus* AF 2525 is from China, AF 2118 is from Indonesia, AF 1305 is from India, AP 2040, AF 2041 and AF 2042 are from Australia, AP 143 is from Uganda and isolates AP 1311, AF 2653, AF 1554, AF 2649 and AF 144 are from USA. Aflatoxigenic isolates are surrounded by circles.

**Figure 5:**
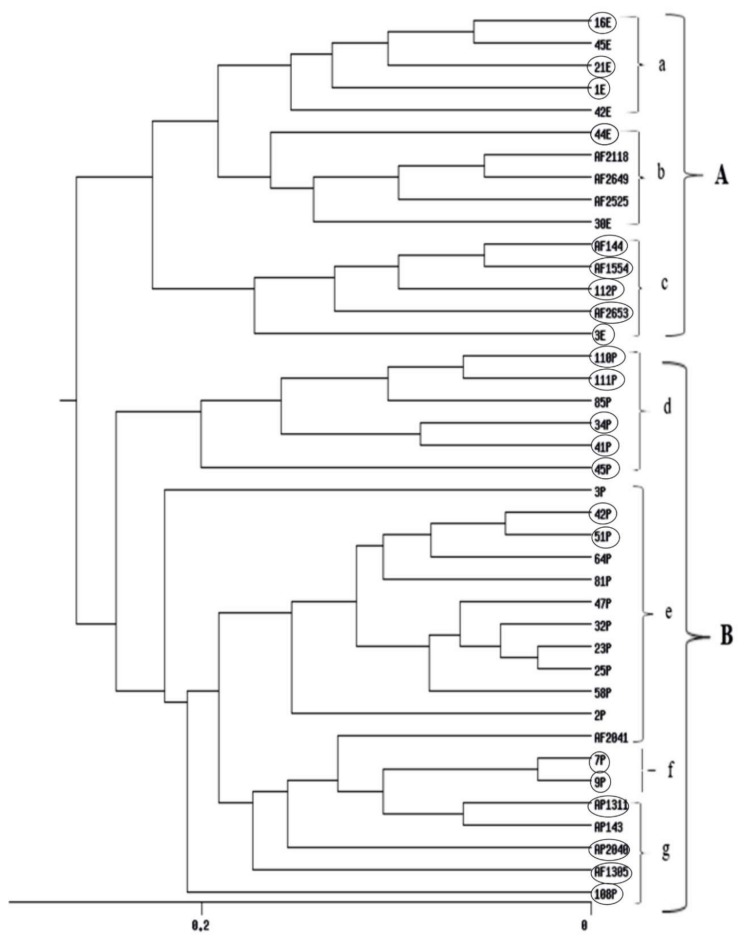
UPGMA cluster analysis generated from ISSR-PCR of 40 *Aspergillus* isolates. E isolates are from Egypt, P isolates are from Philippines, AF 2525 is from China, AF 2118 is from Indonesia, AF 1305 is from India, AP 2040, AF 2041 and AF 2042 are from Australia, AP 143 is from Uganda and isolates AP 1311, AF 2653, AF 1554, AF 2649 and AF 144 are from USA. Aflatoxigenic isolates are surrounded by circles.

UPGMA cluster analyses obtained from RAPD, ISSR and their combined data are shown in (Figure 4, Figure 5 and Figure S2) respectively. The dendrogram produced by RAPD obviously separated the data into two main clusters, B comprises of three isolates and Cluster A includes five sub-clusters (a, b, c, d and e). Sub-cluster (a) comprises of 12 *Aspergillus* isolates, sub-cluster (b) includes four isolates, sub-cluster (c) comprises of two isolates, sub-cluster (d), 11 isolates and sub-cluster (e), six isolates. The ISSR-based dendrogram divided the data into two main clusters. Cluster A includes three sub-clusters a, b and c each of what contains five isolates. Cluster B comprises of four sub-clusters d, e, f and g which include 6, 12, 2, and 5 *Aspergillus* isolates respectively. The UPGMA obtained from combining the two molecular markers showed similar relationships for that obtained by separate trees.

## 3. Discussion

One of the most significant strategies to deal with mycotoxin contamination in crops is the study of the molecular genetics, metabolic, and diversity of mycotoxigenic fungi [25]. Understanding the genetic differences between aflatoxigenic and non-aflatoxigenic isolates is of interest for the reason that aflatoxin-nonproducing isolates of *A. flavus* are utilized to control aflatoxin contamination [26,27]. A total number of forty-*Aspergillus* section *Flavi* isolates, isolated from different geographic regions were analyzed for aflatoxin profiling, molecular identification and molecular diversity. The mycotoxin metabolic profiles of the *Aspergillus* isolates in our study was similar to profiles reported in other studies [28,29] which showed production of both B- and G-types aflatoxins for *A. parasiticus* and *A. nomius*, only B-types *A. flavus*, and no aflatoxins for *A. tamarii*.

The ecological conditions in the Philippines, categorized by high temperature and high relative humidity encourage mycotoxigenic fungi growth and mycotoxin production in agricultural crops [30]. Aflatoxin research in the Philippines started in 1967. According to a recently published review [20], seven *Aspergillus* species, *A. flavus, A. parasiticus, A. carbonius, A. japonicus, A. ochraceous, A. niger*, and *A. westerdijkiae* have been identified from the Philippines crops and predominantly aflatoxins are produced by *A. flavus* species. In addition, *A. tamarii* isolates have been found in Philippines by [31]. Our study identified the majority of the isolates from the Philippines, as *A. flavus,* confirmed that *A. tamarii* is present in Philippines and discovered one new species, *A. nomius* from the Philippines. Previously *A. nomius* isolates were identified morphologically as *A. parasiticus* [21]. *A. tamarii* was a non-producing isolate, while *A. nomius* isolates secreted huge quantities of the four aflatoxin types. This increases the health risk factors from consumption of contaminated commodities by humans and animals. 

Many Egyptian researchers are directed to explore fungal contamination and its toxin production in Egyptian crops and commodities. Most of them are emphasized on *A. flavus* which they have investigated in soybean [32], maize, wheat, rice, peanut seeds [33], cotton [24] and sesame [34]. We collected eight *Aspergillus* isolates from maize, soil, bench and air. Based on molecular identification, all of them; were identified as *A. flavus.* Most of them were aflatoxin producers. Based on our results, we confirmed that *A. flavus* is a predominant producer of B-type aflatoxins in Egypt.

The *aflR* gene plays a crucial role in the aflatoxin biosynthesis pathway by regulating transcription for most, if not all, the structural genes of the aflatoxin gene cluster and encodes a protein containing a zinc-finger DNA-binding motif [35]. Earlier study [36] reported that deletion of *aflR* in *A. parasiticus* stops the expression of other aflatoxin pathway genes. In this study, all aflatoxigenic isolates showed amplified *aflR* PCR products while, non- aflatoxigenic isolates produced less or no *aflR* PCR products. Furthermore, the phylogenetic tree for the *aflR* sequences approximately separated most toxin-producing isolates from the non-aflatoxigenic ones. Another study [37] illustrated that regulatory gene, *aflR* can be used as an early indicator for aflatoxin production. Nevertheless, some of our results of *aflR* gene amplification were incompatible with the HPLC aflatoxin analysis. So, it is not constant to differentiate between aflatoxigenic and non-aflatoxigenic isolates by utilizing PCR amplification of AF biosynthesis genes because there are several mutations in the AF cluster genes within the targeted binding site of the primers and this agrees with data reported in the literature [38,39].

In this study, we used RAPD and ISSR molecular markers to assess the genetic diversity levels between *Aspergillus* populations from eight geographic regions around the world (USA, Philippines, Egypt, India, Australia, Indonesia, China and Uganda). Both RAPD and ISSR markers have been successfully employed in phylogenetic and diversity studies as they are simple, inexpensive and only reliant on thermal cycler and gel electrophoresis systems. They sometimes show low reproducible profiles, but this can be overcome by choosing adequate DNA extraction procedure. Although we used only six primers, we got high genetic variation among the isolates. The banding patterns of RAPD and ISSR-PCR products are summarized in two values, Polymorphism Information Content and Marker Index. Polymorphism is the incidence of different forms among the members of a population and if the PIC value is higher than 0.5 this means that the primer is an effective marker for estimating genetic diversity. Our results showed PIC ˃ 0.5 in both RAPD and ISSR primers. However, ISSR primers showed higher average PIC and MI values than the RAPD primers. This is due to their primer length which permits the annealing at higher temperatures leading to higher diversity [18].

Nei’s gene diversity, H was used to measure the genetic varieties within populations in combination with I, the Shannon’s information index, and they have higher values in ISSR than those in RAPD. Moreover, AMOVA analysis was done to determine the differentiation between three populations from the SRRC culture collection, Philippines and Egypt; using molecular markers. It was clear that AMOVA data present high variation within population higher than among the populations in both markers, which are similar to the results obtained by other species using RAPD or ISSR markers [40].

Cluster analysis drawn by using UPGMA showed clear separation patterns within the populations. The dendrogram based on the RAPD profiles divided the data into two main groups A & B. Group B collected the majority of non-toxic *A. parasiticus* isolates. Group A is divided to five subgroups. The majority of subgroup a are aflatoxigenic isolates from the SRRC culture collection. Subgroup b gathered non-aflatoxigenic isolates from the Philippines. Moreover, the two new genotypes 7P and 9P from the Philippines were clustered in subgroup c. Aflatoxigenic isolates from the Philippines were common in subgroup d. The last subgroup e involved aflatoxigenic isolates.

Based on the ISSR-UPGMA tree, the data were divided into two main clusters A and B. Cluster A has 3 subgroups. Major AF-producing isolates from Egypt are collected in subgroup a. Non-aflatoxigenic isolates from the SRRC culture collection are gathered in subgroup b. Subgroup c clustered five aflatoxigenic isolates. Cluster B was subdivided to four subgroups. Subgroups d and e collected isolates from the Philippines nevertheless, Aflatoxigenic were in d and non-aflatoxigenic in group e. Again, the new genotypes, 7P and 9P- identified as *A. nomius* were gathered together in subgroup f. The last subgroup g in cluster B had aflatoxigenic isolates from the SRRC culture collection. Also in the combined RAPD/ISSR UPGMA some groups were connected to geographic origin and species. All isolates of the main cluster A were *A. flavus* isolates from Philippines, while all *A. flavus* isolates of subgroup d of main cluster B were from Egypt. *A. tamarii* isolate was between subgroups of main cluster B. *A. nomius* isolates 7P and 9P formed subgroup b and *A. parasiticus* isolates AP 134 and AP 1311 were in subgroup c of main cluster B. Main cluster A was mainly non-aflatoxigenic, while all subgroups of main cluster B were mainly aflatoxigenic.

## 4. Conclusions

In this study, we successfully discovered new genotypes of *A. nomius* from the Philippines soil samples. A biocontrol strategy should be performed to eliminate aflatoxins formed in Egypt and the Philippine commodities during pre-harvest, harvest, or storage stages. The *afl*R gene is essential for AF production as it was amplified in all AF producing isolates. On the other hand, it also exists in some non-aflatoxigenic isolates. Both RAPD and ISSR molecular markers were enough to provide complete genetic diversity between *Aspergillus* spp. Though, ISSR markers presented higher genetic variables than RAPD markers.

## 5. Materials and Methods

### 5.1. Chemicals, Reagents, and Media

The mycotoxin standard (5 mL) of aflatoxin mix was purchased from Sigma Aldrich, dissolved in methanol. It contains 1 μg/mL (1000 ppb) aflatoxin B1, 1 μg/mL (1000 ppb) aflatoxin G1, 3 μg/mL (3000 ppb) aflatoxin B2 and 3 μg/mL (3000 ppb) aflatoxin G2. Chloroform, acetonitrile, hexane, trifluoroacetic acid and methanol were purchased from Fisher Scientific company (Waltham, MA, USA). TBE buffer (Tris/Borate/EDTA) was prepared in 1L by adding 54 g of Tris base, 27.5 g of boric acid and 20 mL of 0.5 M EDTA. pH was adjusted to 8.3 by HCl. The result buffer is highly concentrated (5X stock solution) so, it was diluted 10 times by ultra-pure water before use. DNA Molecular Weight Marker VI (Roche, 154–2176 bp) (0.25 µg/µL), was diluted with TE buffer and DNA loading dye to get the final concentration is (0.04 µg/µL). In this study we used an alternative dye to Ethidium bromide which is non-carcinogenic, Midori Green Advance DNA Stain was purchased from Nippon Genetics Europe GmbH (Dueren, Germany).

All media used in this study were sterilized using an autoclave (CertoClav) at 121 °C and three bars for 20 min.

Potato dextrose Agar (PDA) was purchased from OXOID and prepared by suspending 39 g in 1L distilled water. Malt Extract broth (ME) was prepared by dissolving 30 g malt extract (MERCK) and 5 g peptone (Fluka) in 1L distilled water. Yeast extract broth (YE) was prepared by dissolving 4 g Yeast extract (aMResco) and 20 g sucrose (VWR CHEMICALS) in 1L distilled water.

### 5.2. Source of Aspergillus Isolates

Eighty single-spore *Aspergillus* isolates were used in this study from different geographic regions around the world. 28 isolates were kindly provided from the SRRC culture collection (Southern Regional Research Centre, New Orleans, USDA, USA) and originally recovered from six countries (USA, India, Australia, Indonesia, China and Uganda). The Egyptian and the Philippines isolates were previously recovered from maize, wheat, peanut and soil samples in our laboratory, molecular biology unit, Turku University, Finland [21]. These eighty isolates were preliminary checked for aflatoxin production (Table S1) and amplified twice by RAPD and ISSR markers but only 40 *Aspergillus* representative isolates from the main groups were selected for more detailed RAPD and ISSR analysis. The isolates were refreshed and showed viable by growing on Potato dextrose agar (PDA). The long term preservation of the isolates was confirmed by [41]. A loop of mycelia and spores were scraped from the sporulating culture and inoculated on 9 cm diameter petri plates and incubated at 25 °C for 7 days. Some of grown *A. flavus*, *A. parasiticus*, *A. nomius*, and *A. tamarii* isolates are shown in Figure S3.

### 5.3. Detection of Aflatoxins (AFB_1_, AFB_2_, AFG_1_, and AFG_2_) using HPLC

*Aspergillus* isolates were screened for their aflatoxin-producing potentials using high performance liquid chromatography (HPLC). Fresh spores from 40 *Aspergillus* isolates were diluted to the desired concentration 1 × 10^6^ spores / ml using 500 µl sterile distilled water while the number of spores was counted under microscope using the hemocytometer (Burker, JH1405-8). The experiments took place upon the inoculation of 50 µl from the spore suspension in an Eppendorf tubes containing 500 µL yeast extract sucrose (YES) broth for 7 days at 25 °C. Three liquid cultures were made as replicates for each isolate. After incubation, the content of each Eppendorf tube was filtered using Whatman No. 1 to discard fungi. For AF extraction, the filtrate was treated three times with 0.5 mL chloroform, followed by vortexing for 30s. The chloroform layer was moved to fresh 1.5 mL Eppendorf tube and evaporated to dryness on a hotplate at 60 °C [42].

Aflatoxin derivatization procedure was applied on the dried Eppendorf tube according to the official method of analysis [43], 200 µL of hexane was added to re-dissolve AF, 50 µL of trifluoroacetic acid was added followed by vortexing for 30 s. The mixture was let to stand for 5 min and 950 µL deionized water: acetonitrile (9:1) was added followed by vortexing for 30 s. The aqueous layer containing aflatoxins was filtered through 0.2 µm syringe filter and stored in dark vials at −20 °C until HPLC analysis. A 500-μL stock solution of AF mix standard in methanol, containing the four types of aflatoxins (G1, B1, G2 and B2), was dried and treated in the same way as the derivatization procedure used for samples.

Derivatized standard and samples were subsequently injected (10 µL) into the HPLC system which was LiChroCART (Agilent Technologies, Waldbronn, Germany) and an Agilent 1100 series device with absorption and fluorescence detectors (Agilent Technologies, Palo Alto, CA, USA). The column was C18 reversed-phase (LiChrospher 100, 125 × 4 mm, 5 µm). The HPLC system was equipped with a UV detector and fluorescence with 365 nm excitation and 464 emission wavelengths. The column temperature was 25 °C. The mobile phase consisted of water: methanol: acetonitrile (60:30:15, *v*/*v*/*v*). The total run time for the separation was approximately 30 min at a flow rate of 500 µL/min [44]. Aflatoxin concentrations were calculated according to the retention times and the areas of the corresponding peaks on the chromatogram using Analyt-FC (Agilent Technologies, Palo Alto, CA, USA) collector.

### 5.4. Molecular Studies of 40 Examined Aspergillus Isolates (Genotypic Analyses)

#### 5.4.1. DNA Extraction

DNA extraction, PCR amplification and sequencing for *Aspergillus* spp. were done in the present study. Conidia of the *Aspergillus* isolates used for the molecular studies were grown on 0.5 mL malt peptone (MP) broth in a 1.5 mL Eppendorf tube. The cultures were incubated at 25 °C for 3–7 d. Grown mycelium was scraped off and about 100 mg mycelium from each isolate was used for genomic DNA extraction. The mycelia were ground into a fine powder using tissue-grinding pestles. The powder was transferred into a 1.5 mL sterile Eppendorf tube and stored at −20 °C until analysis. DNA was extracted from the cells using GenElute^TM^ Plant Genomic DNA Miniprep Kit (purchased from Sigma Aldrich, Darmstadt, Germany) according to the recommendations of the manufacturer. The highly genomic DNA was stored at −20 °C until analysis.

#### 5.4.2. PCR Amplification and Sequencing

The quality of DNA samples from all isolates was tested by amplification with ITS forward and reverse primers [45]. Also, the presence of the transcriptional regulator, *aflR* gene-participating in aflatoxin biosynthesis pathway was examined in 12 isolates. Amplification was done using the primer pairs shown in Table S2 [46,47].

The PCR mixtures were made to a final volume of 25 μL, containing PCR buffer (20 mM Tris-HCl, pH 8.4, 50 mM KCl), 0.25 mM dNTPs, 50 µM of each primer, 2 U Taq DNA polymerase (F-501L, Thermo Scientific) and 25 ng genomic DNA. Amplification of the gene sequence of each isolate was carried out in a PTC-200 DNA DNA Engine Thermal cycler (MJ Research, Inc.), and these amplified products were purified using Amicon^®^ Ultra-0.5 Centrifugal Filter Devices (Merck, Darmstadt, Germany) according to the guide procedure. After separation on 1% agarose gel, size of the bands was estimated using 100 bp DNA Marker (150–2100 bp). The amplified ITS products from 26 isolates and *aflR* gene products from 14 isolates were sequenced by the FIMM Technology Centre, Helsinki, Finland.

#### 5.4.3. RAPD and ISSR Amplifications

A total of 3 RAPD and 3 ISSR primers were used for DNA amplification. RAPD amplification was performed with RAPD 1, RAPD 2 and RAPD 5 primers. ISSR amplification was performed with (GTG) 5, (GACA) 4, and (AGAG) 4G primers. The primer sequences are shown in Table S2 [48,49]. These primers were selected because earlier [23] reported that these six primers have high reproducibility and clear banding profiles. PCR reactions were performed using a single primer at a time, to a final volume of 25 μL, containing reaction buffer (20 mM Tris-HCl, pH 8.4, 50 mM KCl), 0.25 mM dNTPs, 50 µM of each primer, 2 U Taq DNA polymerase and 25 ng genomic DNA. All PCR products from RAPD and ISSR primers were amplified triplicates and only reproducible results were accepted. Table S3 exhibits the optimization of the thermal cycle of PCR reaction for each primer.

#### 5.4.4. Gel Electrophoresis

The result of each amplification reaction (5 μL) was analysed by electrophoresis in TBE buffer 0.5× (pH = 8.3) in 1% agarose gels and run at 80 V for 45 min, using DNA Molecular Weight Marker VI. Amplified fragments were then visualized and photographed under UV light using the ChemiDoc MP Imaging System (Bio-Rad, Hercules, CA, USA). Size standards were loaded in the first and last wells. The Program GEL (Patzekin and Klopov, Petersburg Nuclear Physics Institute, Gatchina, Russia) was used for analysing gel images.

### 5.5. Phylogenetic Analysis

The average and standard error of aflatoxin quantity were calculated by GraphPad Prism 5 program. The identity of the ITS sequences was specified using Basic Local Alignment Search Tool (BLAST) algorithm in the NCBI (National Center for Biotechnology Information) GenBank database https://blast.ncbi.nlm.nih.gov/Blast.cgi. They were transformed into FASTA format using Molecular Evolutionary Genetic Analysis (MEGA).

Neighbor joining tree for *aflR* sequences of aflatoxigenic and non-aflatoxigenic *Aspergillus* isolates was conducted using MEGA-X software. Number of alleles per locus (Na), effective number of alleles (Ne), Nei’s gene diversity (He), Shannon’s diversity index (I) were determined with the help of POPGENE software (version 1.32). Analysis of molecular variance (AMOVA) was determined using GenAlex software ver. 6.5. Polymorphism Information Content (PIC) value was calculated by the help of Molkin 3.0 software. Marker Index (MI) was calculated according to: https://irscope.shinyapps.io/iMEC/ [50]. RAPD and ISSR dendrograms were constructed by insilico online program http://insilico.ehu.es/dice_upgma/.

## Figures and Tables

**Table 1:** *Aspergillus* section *Flavi* isolates used in this study, toxin production, and molecular identification.

Geographic Origin	Source	Sample ID	Identification	GenBankaccession Number/Identification Sequence Type	Identity	Aflatoxin Production (ppb)				
						AFG1	AFB1	AFG2	AFB2	TotalAFs
**SRRC culture collection**	Cottonseed, USA	AF 144	*A. flavus*	MH752568/*aflR* gene	529/529 (100%)	ND	30.68 ± 25.39	ND	ND	30.68
	Karnataka, India	AF 1305	*A. flavus*	KF432854/ITS	535/535 (100%)	ND	67.5 ± 0.12	ND	0.9 ± 0.45	68.4
	Pistachio, USA	AF 1554	*A. flavus*	MH752566/*aflR* gene	516/516 (100%)	ND	40.38 ± 34.1	ND	ND	40.38
	Peanut, Australia	AF 2041	*A. flavus*	MH244421/ITS	537/537 (100%)	ND	ND	ND	ND	ND
	Dried fish, Indonesia	AF 2118	*A. flavus*	MN511750/ITS	-	ND	ND	ND	ND	ND
	Dead termites, China	AF 2525	*A. flavus*	FN398160/*aflR* gene	525/525 (100%)	ND	ND	ND	ND	ND
	Lung tissue, USA	AF 2649	*A. flavus*	AY510451/*aflR* gene	528/528 (100%)	ND	ND	ND	ND	ND
	Corneal ulcer, USA	AF 2653	*A. flavus*	KY630136/*aflR* gene	525/525 (100%)	ND	3.585 ± 0.03	ND	2336 ± 6.48	2339.6
	Peanuts, Uganda, Africa	AP 143	*A. parasiticus*	MN511749/ITS	-	ND	ND	ND	ND	ND
	Rice, USA	AP 1311	*A. parasiticus*	KC769508/*aflR* gene	526/526 (100%)	157.8 ± 2.57	559.1 ± 22.35	1422 ± 17.20	171.9 ± 3.315	2310.8
	Peanut, Australia	AP 2040	*A. parasiticus*	MH752575/*aflR* gene	524/524 (100%)	903.4 ± 17.49	4.755 ± 0.17	23.50 ± 0.47	ND	931.7
**Philippines**	Soil	2P	*A. tamarii*	MN511748/ITS	-	ND	ND	ND	ND	ND
	Soil	3P	*A. flavus*	LN482489/ITS	539/539 (100%)	ND	ND	ND	ND	ND
	Soil	7P	*A. nomius*	MH752557/ITS	505/505 (100%)	3549 ± 28.3	315.2 ± 15.47	777.5 ± 7.48	72.53 ± 2.03	4714.23
	Soil	9P	*A. nomius*	AY510454/ITS	512/512 (100%)	10705 ± 3.4	1334 ± 4.52	2035 ± 5.36	342.1 ± 20.33	14,416.1
	Soil	23P	*A. flavus*	KX426971/ITS	536/536 (100%)	ND	ND	ND	ND	ND
	Soil	25P	*A. flavus*	MN511747/ITS	-	ND	ND	ND	ND	ND
	Soil	32P	*A. flavus*	KF432854/ITS	535/535 (100%)	ND	ND	ND	ND	ND
	Soil	34P	*A. flavus*	KY630136/*aflR* gene	528/528 (100%)	ND	13.28 ± 2.87	ND	ND	13.28
	Soil	41P	*A. flavus*	MF094441/*aflR* gene	524/524 (100%)	ND	32.89 ± 12.9	ND	ND	32.89
	Soil	42P	*A. flavus*	MG720232/*aflR* gene	524/524 (100%)	ND	6.614 ± 1.4	ND	ND	6.614
	Soil, peanuts	45P	*A. flavus*	FN398161/*aflR* gene	475/475 (100%)	ND	51.04 ± 22.59	ND	ND	51.04
	Soil	47P	*A. flavus*	MH595954/ITS	537/537 (100%)	ND	ND	ND	ND	ND
	Soil	51P	*A. flavus*	FN398157/*aflR* gene	529/529 (100%)	ND	5.058 ± 1.11	ND	ND	5.058
	Soil	58P	*A. flavus*	MK791661/ITS	511/511 (100%)	ND	ND	ND	ND	ND
	Soil	64P	*A. flavus*	KX426971/ITS	536/536 (100%)	ND	ND	ND	ND	ND
	Soil	81P	*A. flavus*	MN511746/ITS	-	ND	ND	ND	ND	ND
	Soil	85P	*A. flavus*	LN482481/ITS	538/538 (100%)	ND	ND	ND	ND	ND
	Soil, coconut	108P	*A. flavus*	MN511745/ITS	-	ND	ND	ND	68.28 ± 20.25	68.28
	Soil, Coconut	110P	*A. nomius*	MN511744/ITS	-	ND	87.50 ± 10.58	204.8 ± 22.29	ND	292.3
	Soil	111P	*A. flavus*	KU561938/ITS	49/51 (94%)	ND	5.055 ± 0.59	ND	ND	5.055
	Peanuts	112P	*A. flavus*	MN511743/ITS	-	ND	58.01 ± 2.57	ND	14.11 ± 1.08	72.12
**Egypt**	Maize	1E	*A. flavus*	MN511742/ITS	-	ND	37.71 ± 13.77	ND	ND	37.71
	Maize	3E	*A. flavus*	MH752568/*aflR* gene	531/531 (100%)	ND	658.1 ± 66.20	ND	114.1 ± 14.69	772.2
	Maize	16E	*A. flavus*	JF729324/ITS	529/529 (100%)	ND	91.06 ± 13.64	ND	29.94 ± 1.3	121
	Maize	21E	*A. flavus*	MG554234/ITS	547/547 (100%)	ND	844.0 ± 15.41	ND	313.6 ± 12.6	1157.6
	Soil	30E	*A. flavus*	MH595954/ITS	535/535 (100%)	ND	ND	ND	ND	ND
	Soil	42E	*A. flavus*	MH595954/ITS	531/531 (100%)	ND	ND	ND	ND	ND
	Bench sample	44E	*A. flavus*	FN398156/*aflR* gene	526/526 (100%)	ND	ND	ND	66.16 ± 9.06	66.16
	Air sample	45E	*A. flavus*	MH595954/ITS	532/532 (100%)	ND	ND	ND	ND	ND

Aflatoxin concentration (ppb) was calculated by the average of three replicate cultures per isolate and expressed as mean ± SEM (Standard Error of the Mean), ND = Not Detectable, PPb = Part Per billion, SRRC = Southern Regional Research Center, New Orleans, LA, USA.

**Table 2:** Details of banding pattern revealed through random amplified polymorphic DNA (RAPD) and inter-simple sequence repeats (ISSR) primers.

	Number of Bands	Number of Polymorphic Bands	PPB (%)	PIC Value	MI
RAPD markers					
RAPD 1	7	6	85.7	0.76	0.55
RAPD 2	5	5	100	0.75	0.48
RAPD 5	5	3	60	0.45	0.39
Average	5.67	4.67	81.9	0.65	0.47
ISSR markers					
(GTG) 5	6	4	66.7	0.77	0.57
(GACA) 4	7	5	71.4	0.79	0.55
(AGAG) 4G	8	8	100	0.81	0.56
Average	7	5.67	79.37	0.79	0.56

PPB = The percentage of polymorphic bands, PIC = The Polymorphism Information Content and MI = Marker Index.

**Table 3:** Analysis of genetic parameters based on ISSR and RAPD markers.

Marker	Number of Alleles (Mean Na)	Effective Number of Alleles (Mean Ne)	Nei’s Gene Diversity (Mean H)	Shannon’s Diversity Index (Mean I)
RAPD	2.00	1.13	0.11	0.223
ISSR	2.00	1.33	0.24	0.408

**Table 4:** Analysis of molecular variance (AMOVA) based on RAPD and ISSR markers from three populations of *Aspergillus* section *Flavi*.

Marker	Source	Df	Ss	Var	%	*P*-Value
RAPD	Among Pops	2	27.342	0.562	8%	0.076 ns
	Within Pops	37	252.433	6.823	92%	0.001 *
	Total	39	279.775	7.385	100%	
ISSR	Among Pops	2	54.423	1.516	15%	0.148 ns
	Within Pops	37	323.777	8.751	85%	0.001 *
	Total	39	378.200	10.267	100%	

Level of significance based on 999 permutations. Df = Degrees of freedom, SS = Sum of Squares, var = Variance component, % = Percentage of total variance.. Ns = non significant, * = significant.

## Supplementary Materials

The following are available online at https://www.mdpi.com/2072-6651/12/1/56/s1, Figure S1: Molecular phylogenetic tree of ITS region for 23 *Aspergillus* section *Flavi* isolates, Figure S2: UPGMA cluster analysis generated from combined RAPD and ISSR data of 40 *Aspergillus* isolates, Figure S3: Isolates of (A) *A. flavus* isolate AF 38, (B) *A. parasiticus* isolate AP143, (C) *A. nomius* isolate 7P and (D) *A. tamarii* isolate 2P grown on PDA after six days of incubation Table S1: Quantification of aflatoxins types using HPLC for 40 *Aspergillus* section *Flavi* isolates, Table S2: Nucleotide sequences of the primers used in this study to amplify the 5′- and 3′-flanks DNA isolated from *Aspergillus* isolates and Table S3: Thermal cycle of PCR amplification for each region & gene.
