# Supplementary material for: A Polyphasic Approach to Compare the Genomic Profiles of Aflatoxigenic and Non-Aflatoxigenic Isolates of Aspergillus Section Flavi
Source: Toxins (Basel). 2020 Jan 16;12(1):56. doi: 10.3390/toxins12010056 (PMC7020436; doi:10.3390/toxins12010056)
Supplement: Supplementary file 1 [file toxins-12-00056-s001.pdf]

Supplementary Materials: A polyphasic approach to compare the genomic profiles of aflatoxigenic and non-aflatoxigenic *Aspergillus* section *Flavi*

Asmaa Abbas, Taha Hussien and Tapani Yli-Mattila

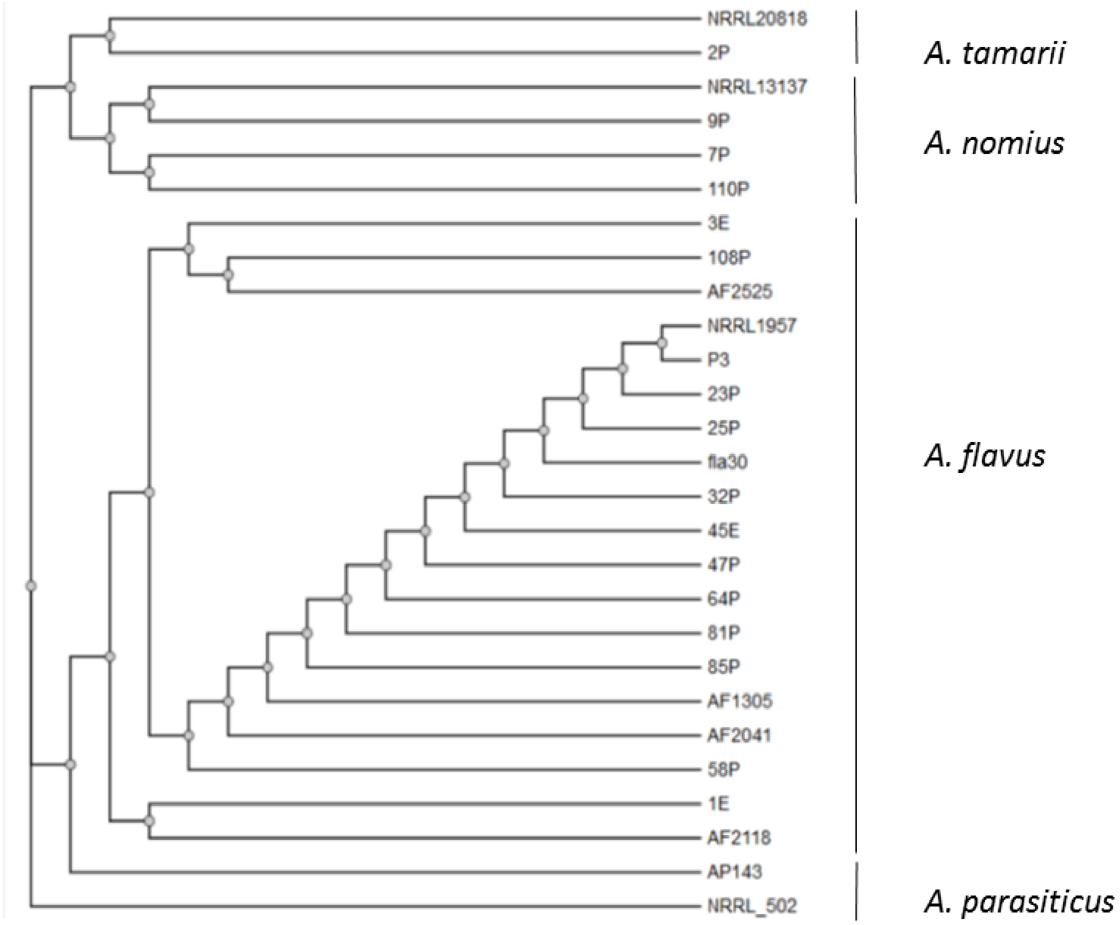

Figure S1. Molecular phylogenetic tree of ITS region for 23 *Aspergillus* section *Flavi* isolates. NRRL13137 is reference strain for *Aspergillus nomius*, NRRL20818 is reference strain for *A. tamarii*, NRRL 502 is reference strain for *A. parasiticus* and NRRL1957 is reference strain for *A. flavus* isolate. The tree was made by using the program of Dereeper A., Guignon V., Blanc G., Audic S., Buffet S., Chevenet F., Dufayard J.F., Guindon S., Lefort V., Lescot M., Claverie J.M. and Gascuel O. Phylogeny.fr: robust phylogenetic analysis for the non-specialist. Nucleic Acids Res. 2008 Jul 1;36(Web Server issue):W465-9. Epub 2008 Apr 19.

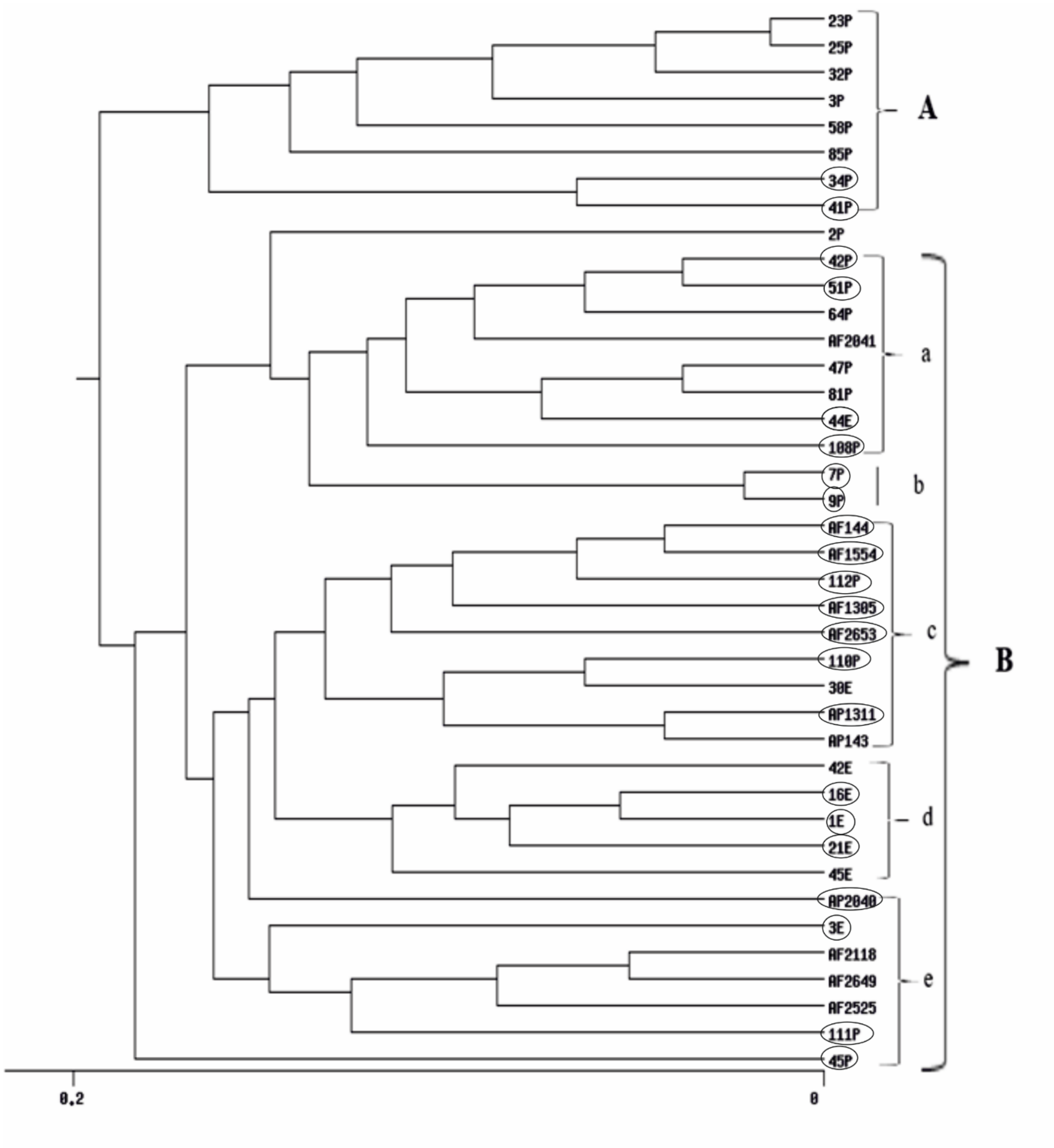

Figure S2. UPGMA cluster analysis generated from combined RAPD and ISSR data of 40 *Aspergillus* isolates. E isolates are from Egypt, P isolates are from Philippines, AF 2525 is from China, AF 2118 is from Indonesia, AF 1305 is from India, AP 2040, AF 2041 and AF 2042 are from Australia, AP 143 is from Uganda and isolates AP 1311, AF 2653, AF 1554, AF 2649 and AF 144 are from USA. Aflatoxigenic isolates are surrounded by circles.

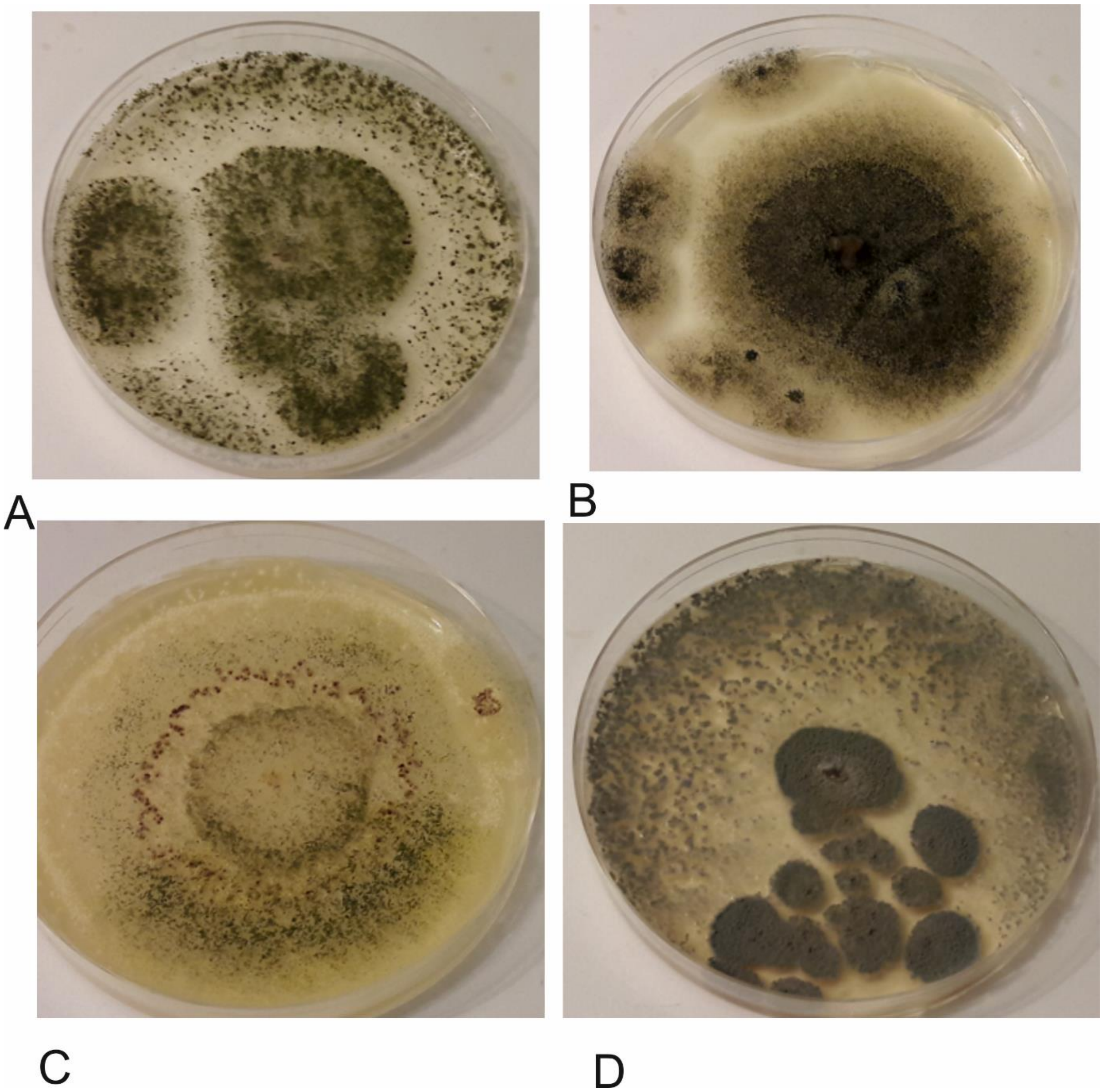

Figure S3. Isolates of (**A**) *A. flavus* isolate AF 38, (**B**) *A. parasiticus* isolate AP143, (**C**) *A. nomius* isolate 7P and (**D**) *A. tamarii* isolate 2P grown on PDA after six days of incubation.

Table S1. Quantification of aflatoxins types using HPLC for 40 *Aspergillus* section *Flavi* isolates.

Isolate Name Egypt	AFG1	AFB1	AFG2	AFB2	Total AF (PPb)
23E	ND	260.1±13.65	ND	547.7±15.75	807.8
29E	ND	1296± 12.58	ND	163.5± 3.915	1459.5
35E	ND	480.4±13.18	ND	112.9±6.71	593.3
40E	ND	11295±38.46	ND	93.56±4.005	11388.56
41E	ND	208.7± 66.38	ND	7.286± 1.591	215.99
43E	ND	572.8± 11.38	ND	321.9± 23.49	894.7
Philippines					
13P	ND	ND	ND	ND	ND
19P	ND	ND	ND	ND	ND
33P	ND	ND	ND	ND	ND
35P	ND	ND	ND	ND	ND
36P	ND	ND	ND	ND	ND
38P	ND	ND	ND	ND	ND
48P	ND	ND	ND	ND	ND

59P	ND	123.5±31.38	ND	15.77±1.619	139.27
62P	ND	ND	ND	ND	ND
63P	ND	ND	ND	ND	ND
69P	ND	ND	ND	ND	ND
77P	ND	ND	ND	ND	ND
86P	ND	ND	ND	ND	ND
90P	ND	ND	ND	ND	ND
96P	ND	ND	ND	85.33±15.14	85.33
107P	ND	ND	ND	1494±103.6	1494
109P	ND	374.2±20.36	ND	201.5±33.14	575.7
SRRC isolates					
AF38	ND	ND	ND	ND	ND
AF283	ND	ND	ND	ND	ND
AF295	ND	ND	ND	ND	ND
AF1356	ND	ND	ND	ND	ND
AF1357	ND	ND	ND	ND	ND
AF1540	ND	ND	ND	ND	ND
AF1550	ND	ND	ND	ND	ND
AF2038	ND	ND	ND	ND	ND
AF2105	ND	ND	ND	ND	ND
AF2112	ND	ND	ND	ND	ND
AF2113	ND	ND	ND	ND	ND
AF2115	ND	ND	ND	ND	ND
AF2632	ND	ND	ND	ND	ND
AP301	ND	ND	ND	ND	ND
AP1310	ND	ND	ND	ND	ND
AP2043	ND	ND	ND	ND	ND
AP2642	ND	ND	ND	ND	ND

Aflatoxin concentration (ppb) was calculated by the average of three replicate cultures per isolate and expressed as mean ± SEM (Standard Error of the Mean), ND= Not Detectable, PPb= Part Per billion, E= Egypt, P= Philippines, AF= *Aspergillus flavus* and AP= *Aspergillus parasiticus*.

Table S2. Nucleotide sequences of the primers used in this study to amplify the 5′- and 3′- flanks DNA isolated from *Aspergillus* isolates.

Primer Code PCR	Target Gene & Region	Sequence 5′– 3′	Product Size (bp)	References
**ITS primers**				
ITS1-F<br>ITS4-R	ITS1 – 5.8S<br>ITS2 - 28S	CTTGGTCATTTAGAGGAAGTAA<br>TCCTCCGCTTATTGATATGC	700	[46]
**RAPD primers**				
RAPD 1<br>RAPD 2<br>RAPD 5	Fragments from 1-10 genomic sites	GGT GCG GGA A<br>GTT TCG CTC C<br>AAC GCG CAA C	298 - 2176	[48]
**ISSR primers**				
(GTG)5<br>(GACA)4<br>(AGAG)4G	Minisatellite-region DNA	GTG GTG GTG GTG GTG<br>GAC AGA CAG ACA GAC A<br>AGA GAG AGA GAG AGA GG	298 - 2176	[49]
**Aflatoxin biosynthesis gene**				
aflR-F<br>aflR-R	*aflR* gene	GGGATAGCTGTACGAGTTGTGCCAG<br>TGGKGCCGACTCGAGGAAYGGGT	650-700	[47]

Table S3. Thermal cycle of PCR amplification for each region & gene.

Region & Gene	Initial Denaturation		Denaturation		Annealing		Elongation		Final Elongation		Cycles Number
	**Temp**	**Time**	**Temp**	**Time**	**Temp**	**Time**	**Temp**	**Time**	**Temp**	**Time**	
ITS	94 °C	3 min	94 °C	30 secs	55 °C	45 secs	72 °C	1 min	72 °C	7 min	35
RAPD	95 °C	5 min	95 °C	1 min	36 °C	1 min	72 °C	2 min	72 °C	5 min	45
ISSR	93 °C	5 min	93 °C	20 secs	55 °C	45 secs	72 °C	90 s	72 °C	6 min	40
aflR	94 °C	3 min	94 °C	30 secs	55 °C	45 secs	72 °C	1 min	72 °C	7 min	35
